# Computer-Aided Diagnosis System of Fetal Hypoxia Incorporating Recurrence Plot With Convolutional Neural Network

**DOI:** 10.3389/fphys.2019.00255

**Published:** 2019-03-12

**Authors:** Zhidong Zhao, Yang Zhang, Zafer Comert, Yanjun Deng

**Affiliations:** ^1^Hangdian Smart City Research Center of Zhejiang Province, Hangzhou Dianzi University, Hangzhou, China; ^2^School of Communication Engineering, Hangzhou Dianzi University, Hangzhou, China; ^3^Department of Computer Engineering, Bitlis Eren University, Bitlis, Turkey; ^4^College of Electronics and Information, Hangzhou Dianzi University, Hangzhou, China

**Keywords:** computer-aided diagnosis system, fetal heart rate signal, recurrence plot, convolutional neural network, optimization experiment

## Abstract

**Background:** Electronic fetal monitoring (EFM) is widely applied as a routine diagnostic tool by clinicians using fetal heart rate (FHR) signals to prevent fetal hypoxia. However, visual interpretation of the FHR usually leads to significant inter-observer and intra-observer variability, and false positives become the main cause of unnecessary cesarean sections.

**Goal:** The main aim of this study was to ensure a novel, consistent, robust, and effective model for fetal hypoxia detection.

**Methods:** In this work, we proposed a novel computer-aided diagnosis (CAD) system integrated with an advanced deep learning (DL) algorithm. For a 1-dimensional preprocessed FHR signal, the 2-dimensional image was transformed using recurrence plot (RP), which is considered to greatly capture the non-linear characteristics. The ultimate image dataset was enriched by changing several parameters of the RP and was then used to feed the convolutional neural network (CNN). Compared to conventional machine learning (ML) methods, a CNN can self-learn useful features from the input data and does not perform complex manual feature engineering (i.e., feature extraction and selection).

**Results:** Finally, according to the optimization experiment, the CNN model obtained the average performance using optimal configuration across 10-fold: accuracy = 98.69%, sensitivity = 99.29%, specificity = 98.10%, and area under the curve = 98.70%.

**Conclusion:** To the best of our knowledge, this approached achieved better classification performance in predicting fetal hypoxia using FHR signals compared to the other state-of-the-art works.

**Significance:** In summary, the satisfied result proved the effectiveness of our proposed CAD system for assisting obstetricians making objective and accurate medical decisions based on RP and powerful CNN algorithm.

## Introduction

Since the brain of a neonate is easily influenced by the oxygen supply, fetal distress caused by a lack of oxygen may lead to different abnormalities that can be considered to be non-life-threatening or life-threatening during pregnancy and delivery (Tharmaratnam, 2000). Thus, an effective tool is required that can monitor the fetal state in real-time and allow obstetricians to take appropriate measures in a timely manner before there is permanent damage to the fetus when an abnormal situation occurs.

In clinical practice, electronic fetal monitoring (EFM), often also called cardiotocography (CTG), is a common way of monitoring a fetal state for obstetricians during intrauterine life (Menihan and Kopel, 2014). The rationale of EFM relies on the understanding that when normal metabolic processes are interrupted either by a lack of oxygen or an inability to expel end-products, the accumulation of acids may threaten all or a part of the vital functions. It has been well established that fetal well-being has a strong relationship with the placenta, the uterus, and the umbilical cord since it depends on not only the adequate functioning of sources and suppliers of oxygen but also on waste removal mechanisms (Ayres-de-Campos, 2016).

Cardiotocography consists of two simultaneously recorded biophysical signals; that is, fetal heart rate (FHR), which is measured in beats per minute (bpm), and uterine contraction (UC) signals, which are measured in either mmHg or an arbitrary unit. With regard to perinatal care, EFM has become a standard tool for preventing unnecessary interventions as well as detecting symptoms of fetal distress (Kunzel, 2012). FHR traces are assessed visually in agreement with common guidelines, such as the International Federation of Gynecology and Obstetrics (FIGO) guideline (Ayres-de-Campos et al., 2015) by obstetricians in clinical practice. However, due to the non-linearity and complexity of fetal dynamics, this visual examination causes high inter- and even intra-observer variability among clinicians (Rhose et al., 2014). Additionally, a false positive is referred to as one of the causes behind the increase in the number of Cesarean sections (CSs) (Steer, 2008). As mentioned previously, although FHR has several drawbacks, it continues to be practiced as a primary diagnostic test in obstetric clinics. To ensure more consistent interpretations of the FHR signal, two basic approaches have been proposed in the literature: extensive training of clinicians and use of computerized systems for medical decision support (Ayres-de-Campos et al., 2010).

Computerized FHR analysis has been adopted as the most promising way to tackle the drawbacks of visual interpretation. This idea is not novel, and in fact, the early studies in this field date back to before the release of general FIGO guidelines (Visser et al., 1981). The majority of these studies focused on either the detection of basic features reflecting FHR characteristics (Dawes et al., 1982; Mantel et al., 1990; Cesarelli et al., 2009) or emulating what experts do in their visual examination (Dawes et al., 1991; Keith and Greene, 1994). Recently proposed systems have been equipped with advanced signal processing, pattern recognition, and machine learning (ML) techniques to anticipate adverse outcomes (Krupa et al., 2011; Czabanski et al., 2012; Spilka et al., 2012, 2014, 2017; Fanelli et al., 2013; Dash et al., 2014; Xu et al., 2014; Doret et al., 2015; Comert and Kocamaz, 2016; Georgoulas et al., 2017; Comert et al., 2018). This approach has three key stages: preprocessing, feature transformation (feature extraction and selection), and classification, which can be briefly described as follows.

### Signal Preprocessing

Gap detection, interpolation, outlier detection, and detrending are frequently utilized in the preprocessing stage of FHR analysis to achieve reliable signals (Cesarelli et al., 2007; Romano et al., 2013).

### Feature Transform

Feature transforms (also called feature engineering in the ML field) have great importance for signal representation. The basic morphological features such as baseline (Dawes et al., 1982), the number of acceleration and deceleration patterns (Mantel et al., 1990) and variability in the short-term and long-term (Cesarelli et al., 2009) are fundamental parts of the computerized FHR analysis. Additionally, the linear and statistical features coming from the time-domain and frequency-domain are extracted to support the automated analysis (Czabanski et al., 2012; Dash et al., 2014; Spilka et al., 2014). Further, using non-linear parameters (e.g., entropy, complexity, and fractal dimension) in fetal state assessment have been proposed and tested (Spilka et al., 2012; Fanelli et al., 2013; Doret et al., 2015; Comert and Kocamaz, 2016). Recently, an image-based time-frequency (IBTF) feature analysis approach comprised of a combination of short term Fourier transform (STFT) and a gray level co-occurrence matrix (GLCM) have been employed as diagnostic indices for fetal hypoxia detection (Comert et al., 2018). Moreover, transform-based analysis methods, such as a discrete wavelet transform (DWT) (Chrelias et al., 2008) and empirical mode decomposition (EMD) (Krupa et al., 2011), have been used for both noise reduction and feature extraction. Especially, heart rate variability (HRV) has become a valuable resource in FHR signal characterization (Romano et al., 2018). On the other hand, since not all extracted features are valuable for classification, feature selection algorithms and dimensional reduction methods have been utilized to select an optimal feature set to improve the performance, including a genetic algorithm (GA) (Xu et al., 2014), information gain (IG) (Spilka et al., 2012), and principal component analysis (PCA) (Zhang and Zhao, 2017).

### Classification

Last, after signal preprocessing and feature transformation, the computerized systems employed ML algorithms to perform two or multiclass classification tasks and thereby discriminate a pathological fetus from a normal fetus. For example, Czabanski et al. (2012) designed an expert system to predict neonatal pathology using a two-stage analysis method based on weighted fuzzy scoring (WFS) and least square support machine (LS-SVM) and obtained good performance with an accuracy of 92.0%. Comert and Kocamaz (2016) applied an artificial neural network (ANN) and performed a classification task with an accuracy, sensitivity, and specificity of 92.40, 95.89, and 74.75%, respectively.

Clearly, although the previous studies concerning computerized FHR analysis presented good classification performance in assessing the fetal state with an accuracy of 90–95%, the conventional ML method needs to extract informative features and select optimal features, which can then be fed into classifiers. Therefore, this approach requires a heavy workload and detailed physiological information regarding the fetus that may be lost during the entire procedure.

Deep learning (DL) has become a highly useful tool for image processing in recent years (LeCun et al., 2015). Especially, convolutional neural networks (CNNs), which involve many layers, have been found to be quite efficient for most image classification problems (Krizhevsky et al., 2012). A CNN essentially self-learns high-level informative features spontaneously by constructing hidden multilayer networks and training data to improve performance in biomedical data processing (Bursa and Lhotska, 2017). Thus, CNN greatly simplifies the subjectivity of feature extraction and discovers the intricate patterns contained in input data compared to manual feature engineering in traditional ML methods. Due to the attractive advantages, CNNs are extensively utilized in the medical field for the purpose of designing diagnostic tools that automatically assist clinicians (Archarya et al., 2017; Comert and Kocamaz, 2018; Li et al., 2018; Petrozziello et al., 2018). For example, Archarya et al. (2017) designed the CNN structure to diagnosis coronary artery disease using an electrocardiogram (ECG) signal and achieved high accuracy of 95.11% (Archarya et al., 2017). In addition, Comert and Kocamaz (2018) also proposed a novel approach to detect fetal hypoxia based on a deep CNN with transfer learning using the FHR signal and Petrozziello et al. (2018) reported their investigation of long short term memory (LSTM) and CNN in analyzing continuous EFM traces from over 35,000 labors for the prediction of fetal compromise. In this study, motivated by previous research, an end-to-end classification strategy (which means that feature transform procedures are ignored) is performed to determine the fetal state using a CNN algorithm, which possesses the ability to self-learn useful features from the input FHR signals.

The remainder of the paper is organized as follows: Section “Materials and Methods” describes the database and overall methodology of the proposed system; Section “Results” presents the experimental setup, results, and relevant discussion; and last, the study’s conclusions are presented and future research is outlined in Section “Discussion.”

## Materials and Methods

**FIGURE 1:**
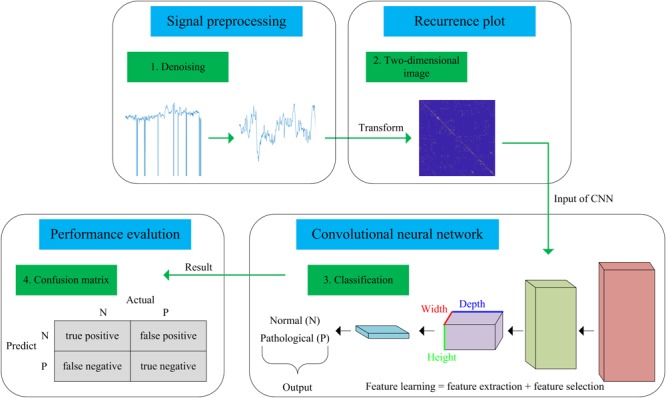
Flow chart of the proposed computer-aided diagnosis (CAD) system incorporating a recurrence plot (RP) and convolutional neural network (CNN).

In this work, we present a novel computer-aided diagnosis (CAD) system aimed at predicting fetal hypoxia based on an advanced DL algorithm. The system diagram of the proposed approach is illustrated in Figure 1. A short description of our approach according to the signal processing flow is given as follows, which can be divided into four steps. First, a relatively pure FHR signal is obtained with a preprocessing algorithm (see Signal Preprocessing). Second, the 1-dimensional time series to 2-dimensional image is transformed using recurrence plot (RP). The ultimate image dataset is expanded by changing the optional parameters of the RP (see Recurrence Plot). Then, based on the enriched data representation, a designed CNN model is applied to learn the intrinsic patterns automatically, which considers the images as input and allows parallel feature self-learning for various characteristics, avoiding time-consuming manual feature engineering (see Convolutional Neural Network). The learned features reflected by the internal parameters of the CNN are then used to enable fetal state assessment. An open-access database is used to test the performance and the pH is chosen as the objective criterion to separate the fetal state into a normal or pathological class (see Database Description). Finally, the classification performance of the proposed system is evaluated using 10-fold cross-validation (see Performance Evaluation).

### Database Description

A publicly accessible intrapartum Czech Technical University - University Hospital in Brno (CTU-UHB) CTG database was used in the experiment. Chudacek et al. (2014) collected the whole signals between 2010 and 2012 using STAN S21/S31 and Avalon FM40/50 EFM devices. All signals were sampled at 4 Hz and stored in electronic form in an OB TraceVue^®^ system provided by Philips. Furthermore, the authors selected a total of 552 intrapartum CTG recordings from a subset of 9164 recordings to constitute this database after considering several technical and clinical criteria, such as the woman’s age, week of gestation, type of gravidity, type of delivery, signal quality, and labor outcome measures. Table 1 shows the main parameters and their respective distributions of this database. Due to a space restriction, interested readers can refer to more detailed information about the database in Chudacek et al. (2014). The maximum duration of the recordings was 90 min and each recording started at a maximum of 90 min before delivery. The database is open access and can be freely downloaded from Physionet (Goldberger et al., 2000).

**Table 1:** Overview of the available information in the open-access intrapartum CTU-UHB cardiotocography (CTG) database.

Information	Mean	Minimum	Maximum
Maternal age (year)	29.8	18	46
Gestational age (week)	40	37	43
pH	7.23	6.85	7.47
BDecf (mmol/L)	4.60	–3.40	
BE	–6.36	–26.80	–0.20
Apgar 1 min	8.26	1	10
Apgar 5 min	9.06	4	10
Gravidity	1.43	1	11
Parity	0.43	0	7
Birth weight (g)	3408	1970	4750
Birth gender		293/259	
(Male/Female)			

All signals were visually interpreted as being divided into four parts by nine experienced obstetricians (Hruban et al., 2015). Moreover, subjective evaluation criteria, Apgar’s scores, were provided for the 1st and 5th min. Conversely, additional biochemical markers measured after delivery, such as the umbilical artery pH, the base excess (BE), and the base deficit in extracellular fluid (BDecf), were provided for an objective categorization. In this work, to make the comparison among different methods more effective (Spilka et al., 2012; Dash et al., 2014; Hruban et al., 2015; Comert et al., 2018), we adopted the suggestion of these studies and the pH value was adjusted to 7.15, which served as a borderline to separate the fetal state into two classes after comprehensive consideration. The signals that had a greater or equal pH than the specified value were assessed as normal. As a result, 447 normal and 105 abnormal (hypoxia) FHR signals were obtained. To avoid the class imbalance problem, we further randomly selected 105 of 447 normal fetuses to keep the number of abnormal fetuses equal.

### Signal Preprocessing

Preprocessing is an important step in biomedical applications and can affect not only the subsequently extracted features but also the final classification performance. The FHR signal has two typical acquisition methods: the Doppler ultrasound probe placed on the abdomen of a pregnant woman externally and the direct ECG measured on an electrode connected to the fetal scalp internally (Kunzel, 2012). From this point of view, the FHR may be contaminated by many aspects of noise, such as the movement of pregnant women and fetuses, improper placement of sensors and other equipment, and external environmental factors. For the sake of content integrity, we briefly describe the preprocessing algorithm used in this work.

**FIGURE 2:**
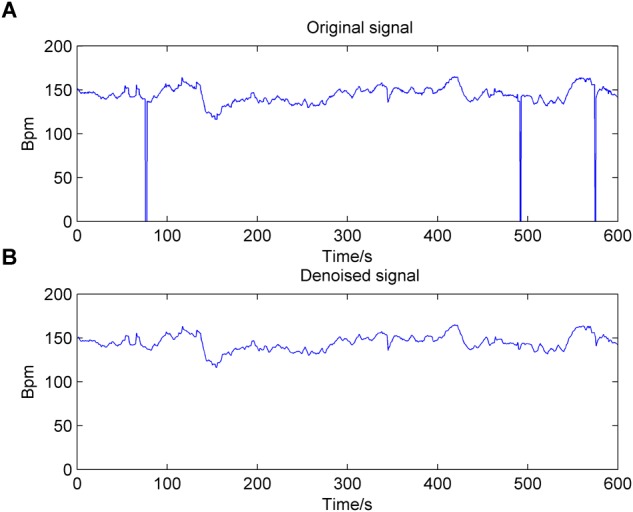
Result of the preprocessing algorithm for the fetal heart rate (FHR) signal used in the work. **(A)**: original signal; **(B)**: denoised signal.

There are two manifestations of noise contained in the FHR signal: missing values and spiky artifacts (Cesarelli et al., 2007). For the former, a signal segment (FHR is equal to 0) that lasts longer than 15 s is removed directly; otherwise, it is linearly interpolated. Then, for the FHR signal that is not stable (the absolute value of two adjacent points is greater than 25 bpm), interpolation is again performed between the initial sampling point and the first point of the next stable part. Finally, the extreme points are also removed; that is, the signal value is greater than 200 bpm or less than 50 bpm, and the segment is then filled in with Hermite spline interpolation. Overall, the preprocessing algorithm can be summarized as having three steps: gap detection, interpolation, and outlier detection (Romano et al., 2013). Figure 2 shows the original noisy signal and preprocessed signal to be further analyzed (10 min in length).

### Recurrence Plot

As a non-stationary and non-linear time series, phase space reconstruction is the first and most important step in the analysis of the FHR signal based on the dynamics theorem. Packard et al. proposed the method of utilizing the time delay coordinates to reconstruct the phase space trajectory of the signal (Packard et al., 1980). Then, Takens (1981) also presented an approach for reconstructing a non-linear system which only requires a time-ordered sequence. Based on Equation (1), a scalar 1-dimensional time series *u_i_* (*i* = 1, 2, 3, …, *L*) is usually embedded into an *m*-dimensional space using this method involving time delay.

(1) $$\begin{array}{c} \begin{array}{c} \begin{array}{c} x=\left\{\begin{array}{c} x_{1}\\ x_{2}\\ .\\ .\\ .\\ x_{k} \end{array}\right\}=\left\{\begin{array}{c} u_{1}\\ u_{2}\\ .\\ .\\ .\\ u_{k+\tau } \end{array}\begin{array}{c} u_{1+\tau }\\ u_{2+\tau }\\ .\\ .\\ .\\ u_{k+\tau } \end{array}\begin{array}{c} ...\\ ...\\ .\\ .\\ .\\ ... \end{array}\begin{array}{c} u_{1+(m-1)\tau }\\ u_{1+(m-1)\tau }\\ .\\ .\\ .\\ u_{k+(m-1)\tau } \end{array}\right\} \end{array} \end{array} \end{array}$$

where the vector *x_k_* represents the *k*-th point on the orbit (*k* = 1, 2, …, L-(m-1)*τ*). The *τ* is the delay time, *m* is the embedding dimension and *m* is ≥2, which are both difficult to be determined.

Furthermore, there is a prior condition in Takens’ theory that assumes that the signal is infinite and does not contain noise, where the delay time *τ* can be chosen almost arbitrarily (Packard et al., 1980). Unfortunately, the real FHR signals recorded by clinical equipment are mostly finite and noisy; therefore, we needed to consider the choice of the delay time carefully. If *τ* is too large, it may incur irrelevance, where the dynamics of an attractor are independent and become causally disconnected. In contrast, if *τ* is too small, it may lead to redundancy, where the reconstructed attractor is compressed along the identity line (Kim et al., 1999).

Similar to the delay time, the embedding dimension vitally needs to be determined. If *m* is too small, the geometry of the phase space is partly folded. If *m* is too large, it may result in massive calculations and increasing the contamination because of the rounding or instrumental error (Kennel et al., 1992). The dimensions of the various portions of the non-linear signal may differ from each other and tend to increase the required dimension influenced by noise. Thus, the choice of a large enough dimension that consists of the relevant dynamics for a noisy signal is considerable (Fraser and Swinney, 1986).

Based on the above-mentioned phase space reconstruction, RP is a qualitative analysis approach for biomedical signals. The concept of RP was proposed by Eckmann et al. (1987), which can depict the recurrence property of a deterministic dynamical system; i.e., visualizing the time dependent behavior of orbits *x_i_* in a phase space. The RP can intuitively reflect the high dimensional phase space motion law of non-linear signals in 2- dimensional space. The key step of an RP is to define the distance between any two vectors in the phase space and its mathematical expression is:

(2) $$\begin{array}{c} R_{i,j}(\varepsilon )=\Theta (\varepsilon -||x_{i}-x_{j}||),i,j=1,...,N \end{array}$$

(3) $$\begin{array}{c} \Theta (x)=\left\{\begin{array}{cc} 1, & x>0\\ 0, & x\leq 0 \end{array}\right\} \end{array}$$

where *𝜀* is a predefined distance threshold; *x_i_* and *x_j_* represent the *i*-th and *j*-th phase space vectors, respectively, which can be reconstructed by using the Takens’ time delay approach mentioned above; *N* is the total number of phase points and *N = L-(m-1)τ*, ||…|| represents the norm (e.g., Euclidean distance), and Θ(x) is a Heaviside function.

We can generally explain Equation (2) as follows: if *x_j_* is sufficiently close to *x_i_*, which means that *x_j_* falls within a ball of the cutoff distance *𝜀* centered at *x_i_, R_i,j_* is 1, then a white dot is placed at a point (*i, j*); otherwise, *R_i,j_* is 0 and the dot is black. Then, the binary values of *R_i,j_* can be simply denoted as the white (1) and black (0) points, respectively. Hence, we can consider the RP as a visual inspection of a high-dimensional phase space trajectory (Eckmann et al., 2008). An *N × N* distance matrix can be converted into a 0-1 matrix and this allows the recursive property of the time series graphically. The *𝜀* is chosen by k’s nearest neighbors method in this work (Ouyang et al., 2014).

**FIGURE 3:**
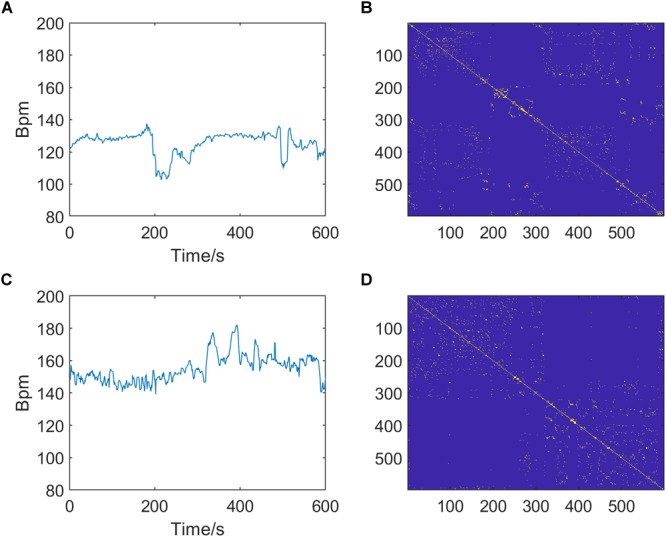
Preprocessed fetal heart rate (FHR) signals (left, **A,C**) and corresponding recurrence plots (RPs, right, **B,D**) of normal (top, **A,B**) and pathological fetuses (bottom, **C,D**). The RP parameters are *m* = 2, *τ* = 1, and *k* = 6.

In summary, there are three optional parameters of RP that can be adjusted: *m, τ*, and *k*, which indicate the embedding dimension, delay time, and distance threshold, respectively. Figure 3 shows the RPs of normal and pathological fetuses.

### Convolutional Neural Network

As a brand-new field that has been developing rapidly for more than a decade, DL has received increasing attention from researchers, and it has obvious advantages over shallow models (i.e., ML) in feature engineering and model building (Zhang and Zhao, 2017). DL is good for excavating an increasing number of abstract features that possess better generalization ability from the original input data, especially the image (Schmidhuber, 2015). It overcomes some of the problems that were thought to be difficult to solve in artificial intelligence (AI) in the past. Moreover, with the significant increase in the number of training datasets and the dramatic increase in chip computational power, DL has achieved remarkable success in object detection, computer vision, natural language processing, and voice recognition, so it has also promoted the development of AI (Deng and Yu, 2014; Bengio, 2015). DL is a hierarchical ML method that includes multilevel non-linear transformations and the deep neural network (DNN) is currently the main form (Canziani et al., 2017). The connection mode between neurons in the DNN is inspired by animal visual cortex organization, and CNN is one of the classical and widely used structures (Bouvrie, 2006). The local connections, weight sharing and pooling operations of the CNN can significantly reduce the complexity of the network. Additionally, the number of training parameters makes the model have a certain degree of invariability to translation, distortion, and scaling (Krizhevsky et al., 2012). In addition, CNN has strong robustness and fault tolerance, and it is also easy to train and optimize. Based on these superior characteristics, CNN effectively outperforms the standard fully connected neural network in various signal and information processing tasks (Bursa and Lhotska, 2017).

**FIGURE 4:**
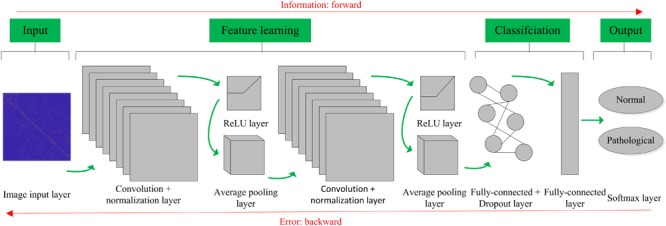
Convolutional neural network (CNN) architecture designed for the proposed computer-aided diagnosis (CAD) system in this work.

The basic structure of the CNN is composed of an input layer, a convolution layer, a pooling layer (also called the subsampling layer), a fully connected layer, and an output layer (Bouvrie, 2006). Convolution and pooling layers generally involve several alternate convolution and pooling layers; that is, one convolution layer is connected to one pooling layer, a pooling layer is followed by one convolution layer, and so on (Liu et al., 2015). Because each neuron of the output feature map in the convolution layer is locally connected to its input, the weighted sum of the corresponding connection weights and local input plus the bias value determine the input value of the neuron. The principle is equivalent to the convolution process in mathematical meaning, and it was therefore given the name CNN. Figure 4 shows a graphical representation of our designed CNN architecture consisting of an image input layer and two convolutions; that is, normalization, ReLU layers, two average pooling layers, two fully connected layers, a dropout layer and a final softmax output layer.

#### Convolution Layer

CNN learns from filters (also referred to as kernels) in the convolution layer, which is an important part of the hidden layers (Bouvrie, 2006). The convolution operation is applied in this layer to the input data, and the extracted features are passed to the next layer, composed of output multiple feature maps. Each feature map is formed by the convolution operation of the convolution kernel for the feature map of the previous layer, expressed as Equation (4). The convolution kernel is the content that the network will learn, including the weight matrix, *W* (i.e., *m*-dimensional filter) and the bias term, *b*. Equation (4) shows that the weight matrix *W* is the same for all neurons in this layer *X^(l)^*, and convolution layers share weights representing learning of the network, which embodies an important feature of CNN. In the convolution layer, the stride, padding factor, number and size of the filters are determined according to the optimization experiment.

(4) $$\begin{array}{c} X^{\left(l\right)}=f(w^{\left(l\right)}⊗X^{\left(l-1\right)}+b^{\left(l\right)}) \end{array}$$

### Activation Layer

After the convolution layer, a batch normalization layer is useful in reducing sensitivity to variations with the input data and an activation layer is usually applied to introduce the non-linearities into the network through mapping to the input data (Krizhevsky et al., 2012). Compared with traditional sigmoid and tanh functions, we employed the rectified linear unit (ReLU) as the activation function because of several attractive advantages (Hara et al., 2015): (i) They possess stronger ability to represent the learned features; (ii) No gradient vanishment phenomenon and the convergence speed of the model is maintained at a stable state; and (iii) The neurons in the neural network have a sparse activation property and the sparse model enables better mining of relevant features and fitting of training data. The form of the function is shown as Equation (5).

(5) $$\begin{array}{c} f(X)=\max(0,WX+b) \end{array}$$

#### Pooling Layer

The pooling layer focuses on a cluster of neurons to reduce the number of weights using max pooling or average pooling operations (Bouvrie, 2006). In this way, the output of a cluster of neurons is represented by a single neuron. Each feature map is downsampled to decrease the dimensions and amount of data, while the important information is retained. This downsampling operation contributes to control overfitting in the learning process along the spatial dimensions, expressed as Equation (6). In the majority of CNN architectures, pooling layers are located among successive convolution layers. The average pooling operation is adopted in this work. In other words, the subsampling function *down(⋅)* calculates the average value of each subset, where the input data are divided into a set of non-overlapping rectangles. In addition, the stride, padding factor, and size of the kernel in the pooling layer are determined according to the optimization experiment.

(6) $$\begin{array}{c} X^{\left(l\right)}=f(Z^{\left(l\right)})=f(W^{\left(l\right)}down(X^{\left(l-1\right)})+b^{\left(l\right)}) \end{array}$$

#### Fully Connected Layer

The output of the convolution and pooling layers described above represent the advanced features extracted from the input images, which are used for classification in the fully connected layer (Akbar et al., 2017). Neurons in this layer have full connections to all activations in the previous layer. The class scores are computed in the fully connected layer. After that, the output of the softmax layer is an *N*-dimensional vector (Rimer and Martinez, 2004), corresponding to the number of classes desired, and *N* is set to two classes (normal and pathological fetuses). In this work, the cross-entropy is adopted as the loss function in the softmax classification layer.

#### Dropout Layer

Overfitting occurs when a model matches better with the training set, rather than the test set; i.e., the training accuracy is high, while the generalization accuracy is low. A dropout layer followed by the fully connected layer in the CNN model prevents overfitting by setting random activations to zero during the training period (Srivastava et al., 2014). The probability of dropout is 0.8 in this work.

### Performance Evaluation

To alleviate the influence of class imbalance, we randomly selected the same number of normal and pathological fetuses, which were both 105. The 1-dimensional preprocessed FHR signal was then transformed into a 2-dimensional RP image. By changing three optional parameters (*m* = 2, 3; *τ* = 1 to 10 in step of 1; *k* = 1 to 10 in step of 1), the ultimate dataset contained 21, 000 images for each class; i.e., 21,000 normal and 21,000 abnormal cases, which were considered to be sufficient for DL training.

**FIGURE 5:**
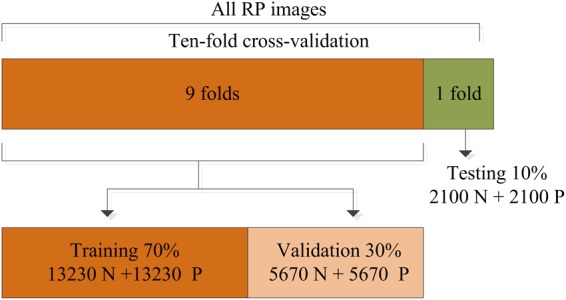
Division of the recurrence plot (RP) image dataset used for training, validation, and testing.

In this work, the training strategy of 10-fold cross-validation was employed in the performance evaluation to obtain more reliable results. The total RP images were separated equally into 10 segments, thereby 9 of 10 images were used in the training and validation of the CNN model while the remainder (1 of 10) of the images were used to test the performance of the proposed system. This process was iterated 10 times by randomly shifting the training data, as shown in Figure 5. The final results recorded in all 10 iterations were averaged and considered to be the overall classification performance.

As with the conventional ML methods, we need some indicators to measure the performance of the proposed system. The confusion matrix is commonly used in a binary classification problem and it consists of four parameters: True Positive (TP), False Positive (FP), False Negative (FN), and True Negative (TN). The literal meaning of above mentioned positive and negative terms denote normal and hypoxia fetuses, respectively. In clinical practice, the accuracy (Acc), sensitivity (Se) and specificity (Sp) are specifically employed to assess the classification algorithms, as shown in Equations (7) to (9). The Se points out the performance of the model on the detection of normal fetus whereas the Sp measures the performance of the system on detecting a hypoxic fetus. In addition, the area under (AUC) the receiver operating characteristic (ROC) curve is also useful for obstetricians.

(7) $$\begin{array}{c} Accuracy=\frac{TP+TN}{TP+FP+TN+FN} \end{array}$$

(8) $$\begin{array}{c} Sensitivity=\frac{TP}{TP+FN} \end{array}$$

(9) $$\begin{array}{c} Specificity=\frac{TN}{FP+TN} \end{array}$$

## Results

### Experiment One: Optimization of the CNN Parameters

In this study, we trained our CNN model on a workstation with an Intel Core 3.70 GHz (i3-4170) processor and a 4GB RAM. The entire process of the proposed approach was implemented with MATLAB^®^ (2018a).

**FIGURE 6:**
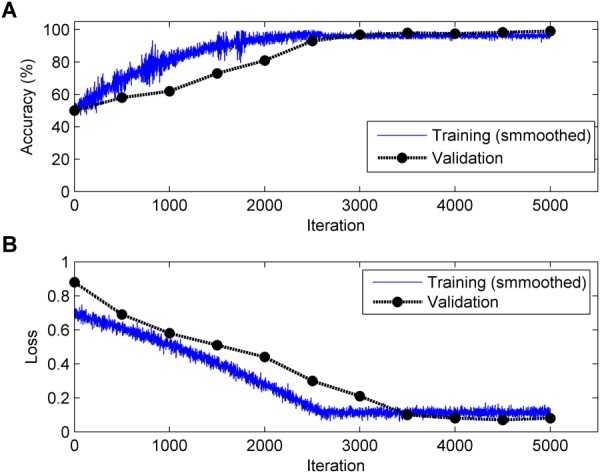
Change in accuracy (top, **A**) and loss (bottom, **B**) during the training and validation processes (Note: 1 epoch = 500 iterations and the validation process is carried out for each epoch).

It is generally acknowledged that the CNN algorithm has many tuning parameters that can influence the classification performance to different degrees. In this work, the initial learning rate was set to 1 × 10^-3^, which controls the relatively stable learning speed. L2 regularization was applied to overcome overfitting with a factor of 1 × 10^-4^. The training and validation process of the CNN model is presented in Figure 6. It can be clearly observed that, as the iteration (epoch) progresses, the Acc increases and the loss decreases for both training and validation. To obtain better results, an optimization experiment was employed using the validation set in this work divided into three primary aspects.

**FIGURE 7:**
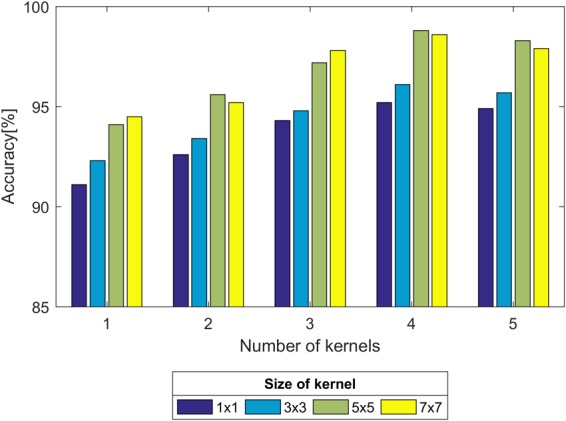
Comparison of the validation accuracy with the size and number of kernels.

First, we experimented with the effect of different layer parameters on performance, which were adjusted in turn. For the convolution layer of the CNN model, the size and number of kernels had a certain relationship with the classification performance, as shown in Figure 7. Based on the validation set, we discovered that the Acc of the size of the convolution kernel of 5 × 5 and 7 × 7 remained approximately the same but was higher than that of 1 × 1 and 3 × 3. The validation Acc increased with the number of kernels before reaching 8. Thereby, we set the size and number of kernels to be 5 × 5 and 8 for higher Acc and less training time (as indicated in Figure 7), respectively. In addition, we found that the parameters of the stride and padding factor in the convolution layer, the stride, padding factor and size of pooling kernel in the pooling layer made the Acc basically stable. In summary, Table 2 presents the determined parameters for each specific layer corresponding to Figure 4.

**Table 2:** Details of the convolutional neural network (CNN) architecture for the proposed computer-aided diagnosis (CAD) system.

Layer	Type	Size	Input			Output
			Number	Stride	Padding	Feature map
0–1	Image input	–	–	–	–	64 × 64 × 3
1–2	Convolution	5 × 5	8	1	0	60 × 60 × 8
2–3	Average pooling	3 × 3	–	2	0	29 × 29 × 8
3–4	Convolution	5 × 5	8	1	0	25 × 25 × 8
4–5	Average pooling	3 × 3	–	2	0	12 × 12 × 8
5–6	Fully connected	144	–	–	–	144 × 1
6–7	Fully connected	2	–	–	–	2 × 1
7–8	Classification output	–	–	–	–	2 × 1

*The normalization and ReLU layers after each convolution layer and the dropout layer located in two successive fully connected layers are not shown above, since they do not change the output feature map.*

**FIGURE 8:**
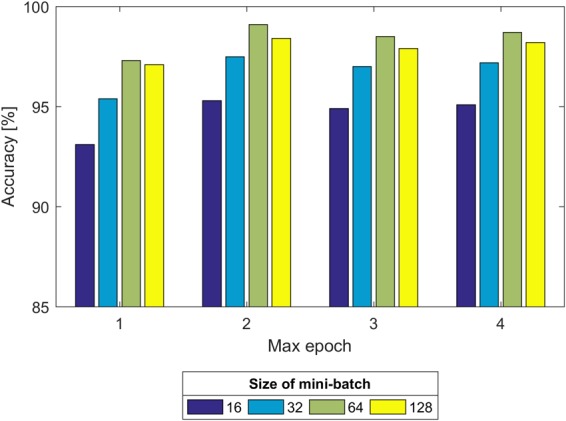
Comparison of the validation accuracy with the size of the mini-batch and max epoch.

Second, we experimented with the effect of size of the mini-batch and max epoch on the training process, as shown in Figure 8. It can be seen that regardless of the max epoch, the validation Acc was higher when the size of the mini-batch was equal to 64. On the other hand, we observed the trained CNN model might not fully learn the image information when the max epoch was 5. Once the max epoch reached 10, the model learned as many features as possible about the fetal state from the input data and the Acc remained essentially constant with increases of the max epoch. Thus, the size of the mini-batch and max epoch were selected as 64 and 10 (as indicated in Figure 8), respectively.

Third, the training of kinds of multilayer neural networks requires the back propagation (BP) algorithm to train the feed-forward neural network better and faster and to obtain the weight and bias parameters of each layer of the neural network. In recent years, researchers have proposed several powerful BP algorithms for the training of a CNN, including stochastic gradient descent (SGD), adaptive moment estimation (ADAM), and root mean square propagation (RMSP). Table 3 presents the three algorithms together with individual parameter settings and the corresponding results. It was obvious that the Acc of the ADAM algorithm was higher than the other two.

**Table 3:** Comparison of the validation accuracy with three back propagation (BP) algorithms.

BP algorithm	Input parameter			Output accuracy (%)
	Momentum	Gradient decay factor	Epsilon	
SGD	0.8	–	–	97.6
**ADAM**	**–**	**0.6**	**1 × 10^-6^**	**99.3**
RMSP	–	0.6	1 × 10^-6^	98.4

*The best performance is indicated in bold.*

### Experiment Two: Performance of the CNN Model

**FIGURE 9:**
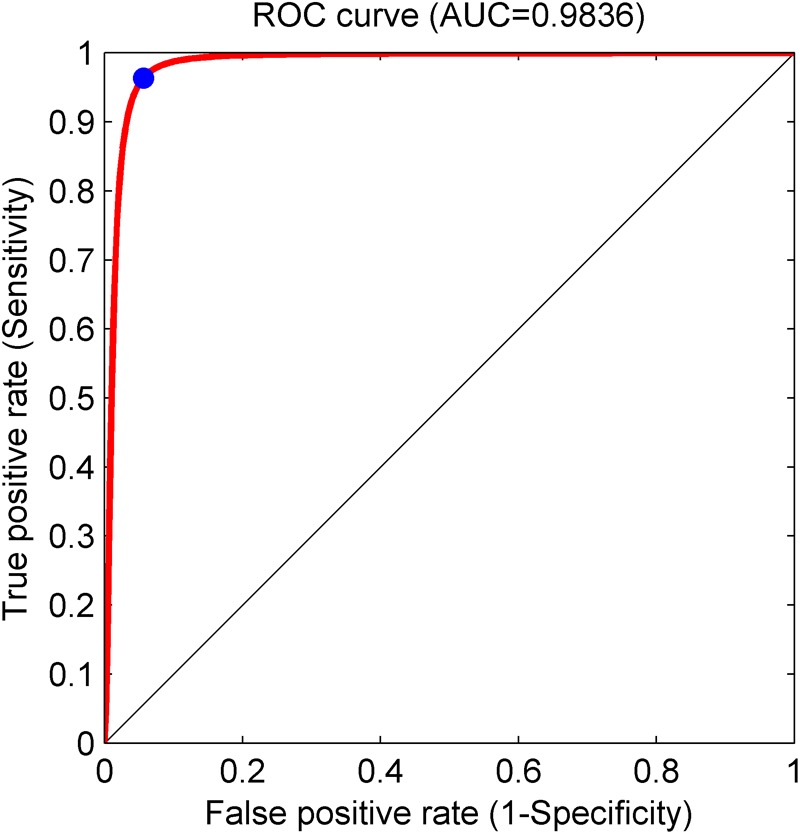
Receiver operating characteristic (ROC) curve with the optimal area under the curve (AUC) of 0.9836 using an 8-layer convolutional neural network (CNN) model. The blue dot represents better performance at the balance of the false positive rate and true positive rate.

Based on the same optimal experimental method described in Experiment 1, we investigated the influence of different layers of the CNN model with their corresponding CNN parameters on the classification performance and Table 4 presents the experimental results using the testing set. Architectures with more than 12 layers demonstrated overfitting or underfitting and therefore were not considered. Clearly, in the beginning, as the number of layers increased, the performance of our proposed system improved. When the number of layers reached 8 layers, consisting of one input layer, two convolution layers, two pooling layers, two fully connected layers, and one output layer (i.e., 13 layers including the normalization and ReLU layers followed by each convolution layer and the dropout layer located in the successive fully connected layers), the best performance was achieved: of 2,100 normal fetuses, only approximately 0.95% (20) were wrongly classified as pathological. Likewise, for hypoxia fetuses, a total of 2.33% (49) were wrongly classified as normal. Moreover, as the number of layers continued to increase, the system performance began to decline instead. Figure 9 shows the ROC curve with the optimal AUC value (CI: 0.9797–0.9875) when the number of layers was 8. In addition, it can be observed from Table 4 that different layers of CNN architecture had approximately the same testing time for one-fold. In other words, once the proposed CNN algorithm was successfully trained, the corresponding CAD system could immediately identify an unknown fetus regardless of the number of layers.

**Table 4:** Comparison of the averaged classification performance with different layers of the convolutional neural network (CNN) model using the test dataset across 10-fold for the proposed computer-aided diagnosis (CAD) system.

Layers: type	TP	FP	FN	TN	Acc (%)	Se (%)	Sp (%)	Time(s)
5: I-C-P-F-O	1884	225	216	1875	89.50	89.71	89.29	25.33
6: I-C -P-C-F-O	1950	138	150	1962	93.14	92.86	93.43	27.51
7: I-C-P-C-P-F-O	2037	86	63	2014	96.45	97.00	95.90	25.37
**8: I-C -P-C-P-F-F-O**	**2080**	**49**	**20**	**2051**	**98.36**	**99.05**	**97.67**	**26.58**
9: I-C-P-C-P-C-F-F-O	2045	68	55	2032	97.07	97.38	96.76	24.71
10: I-C -P-C-P-C-P-F-F-O	2011	75	89	2025	96.10	95.76	96.43	28.29
11: I-C-P-C-P-C-P-F-F-F-O	2063	59	37	2041	97.71	98.24	97.19	27.44
12: I-C -P-C-P-C-P-C-F-F-F-O	2038	63	62	2037	97.02	97.05	97.00	26.13

*I, image input layer; C, convolution + normalization + ReLU layer; P, average pooling layer; F, fully connected + dropout layer; O, classification output layer. The best performance is indicated in bold.*

**FIGURE 10:**
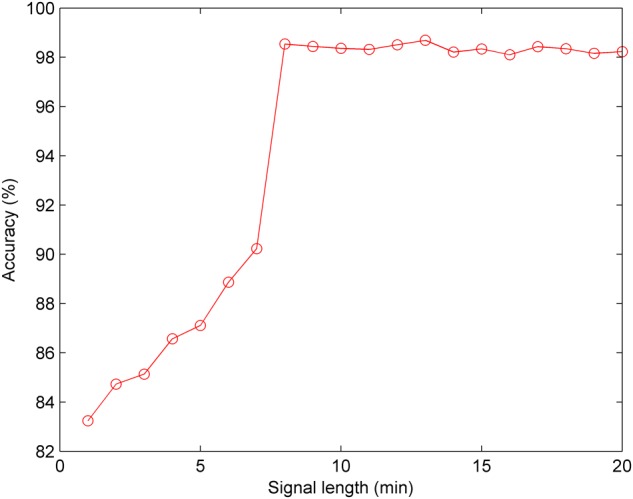
Comparison of the averaged accuracy with different fetal heart rate (FHR) signal lengths using the test set across 10-fold for the proposed computer-aided diagnosis (CAD) system.

Furthermore, we conducted the experiment concerning the impact of the signal length on the classification performance. According to the optimal CNN configuration, Figure 10 shows that when the signal length is less than 8 min, the Acc increases gradually; and when the signal length is greater than 8 min, the Acc fluctuates within a small range. Therefore, we discovered that 8 min was the minimum length of the FHR signal containing relevant information regarding the fetus, and 13 min achieved better performance (Acc = 98.69%) using the CNN for fetal state classification.

## Discussion

Since the vital activities of the fetus, such as nutrition and breathing, directly depend on the placenta, there is a remarkable relationship between them. The key role of the placental function is to ensure the exchange between the fetus and the mother via the umbilical cord, and this cycle is associated with the maternal placental blood flow. This flow is significantly shaken by the tone of the uterine muscle. A contraction over 30 mmHg leads to stopping of the maternal blood flow, and then the fetus encounters with a stressful condition arising from lack of oxygen (Sundstrom et al., 2005). In order to maintain growth and energy production, it is necessary to transport oxygen to the tissues and cells. Depends on the oxygen saturation in the umbilical cord, fetus uses its own defense mechanisms to regulate its physiological condition by balancing its cardiac activities. This in part reflects the physiological resilience of the healthy fetus and this process is monitored via EFM devices (Lear et al., 2018). In a stressful condition arising from oxygen deficiency, the fetus decreases the cardiac activity and energy consumption. A healthy fetus can tolerate to this condition about hours. This stressful condition matches a deceleration pattern on the CTG trace. In addition, several complex physical events such as major placental abruption, uterine rupture, umbilical cord prolapsed, maternal cardiorespiratory disorders, Aorto-Caval compression, etc., may also cause undesired situations (Ayres-de-Campos, 2016). On the other hand, when the tissues and cells are well oxygenated, the fetus increases cardiac activity and consumes energy. This period can be observed as an acceleration pattern on a CTG trace. The variability in short and long terms, as well as acceleration patterns, point fetal well-being whereas deceleration patterns are associated with stressful conditions. Although there is not a gold standard regarding the FHR length in the computerized FHR analysis, observing an acceleration pattern in 15 min is adopted as a healthy condition for the fetus (Ayres-de-Campos et al., 2015). In summary, the interpretation of these physiological complex events on a paper trace is a difficult task. For this reason, it should be cautious during the visual examination, and this process should be supported using numerical approaches in order to ensure a more consistent objective examination.

Continuous EFM is used worldwide to visually evaluate whether a fetus is exhibiting signs of hypoxia during labor, and may benefit from an emergency operative delivery. Previously, computerized EFM assessment that mimics clinical experts showed no benefit in randomized clinical trials (Petrozziello et al., 2018). The current CAD systems utilize some specific morphological features to make a decision about the fetal status according to the common guidelines (e.g., FIGO), including the baseline, number of acceleration and deceleration patterns (Ayres-de-Campos et al., 2015). However, as an example of routinely collected ‘big’ data, EFM interpretation should benefit from data-driven computational approaches, such as ML and DL, which allows automated evaluation based on large clinical datasets (Abdulrahman et al., 2017).

To decrease the incidence rate of the unnecessary CSs caused by subjective diagnostic error, many researchers have proposed different methods for automated assessment of fetal well-being based on advanced AI algorithms, as summarized in Table 5. It can be observed that the previous work employed the same strategy: signal preprocessing, feature extraction and selection, and final classification. In other words, the authors first required to extract and select an optimal set of informative features, which were then fed into classifiers. Therefore, this conventional method has some drawbacks: the feature engineering process is much too complex and sometimes the physiological information about the fetal state may be lost or the used features may be insufficient for FHR classification, which make the performance of fetal state assessment not high (the Acc is less than 94%).

**Table 5:** Summary of related works aimed at the prediction of the fetal state using fetal heart rate (FHR) signals obtained from cardiotocography (CTG).

Author Year	Database	Distribution (N/P)	Method			Performance(%)
			FE	FS	C	
Krupa et al., 2011	Private	Imbalance (30/60)	EMD	/	SVM	Acc: 87 Se: 95 Sp:70
Spilka et al., 2012	Private	Imbalance (123/94)	33 S1, S2, S3	PCA, IG	NB, SVM, DT	Se:73.4 Sp:76.3 Fm:71.5
Czabanski et al., 2012	Private	Imbalance (146/43)	7 S1	/	WFS+ LS-SVM	Acc:92.0 QI:88.2
Fanelli et al., 2013	Private	Balance (61/61)	PRSA	/	ST	AUC = 75
Xu et al., 2014	Private	Balance (255/255)	64 S1, S2, S3	GA	SVM	Se:83 Sp:66 AUC:74
Dash et al., 2014	Private	Imbalance (60/23)	8 S1	/	GM, NB	Se: 61 Sp:82
Spilka et al., 2014	CTU-UHB	Imbalance (175/377)	33 S1, S2, S3	/	LCA+RF	Se:72 Sp:78
Doret et al., 2015	Private	Imbalance (30/15)	12 S2, S3	/	ST	AUC:87
Comert and Kocamaz, 2016	CTU-UHB	Balance (272/280)	11 S2, S3	/	ANN	Acc: 92.40 Se:95.89 Sp:74.75
Georgoulas et al., 2017	CTU-UHB	Imbalance (508/44)	33 S1, S2, S3	AUC	LS-SVM	Se:72.12 Sp:65.30
Spilka et al., 2017	Private	Imbalance (1251/37)	20 S2, S3	/	S-SVM	Se:73 Sp:75
Comert et al., 2018	CTU-UHB	Imbalance (439/113)	IBTF	GA	LS-SVM	Se: 63.45 Sp: 65.88
Comert and Kocamaz, 2018	CTU-UHB	Imbalance (508/44)	STFT+CNN			Se: 56.15 Sp: 96.51
Petrozziello et al., 2018	CTU-UHB	Imbalance (unknown)	LSTM/CNN			AUC: 82
Li et al., 2018	Private	Imbalance (3012/1461)	CNN			Acc: 93.24
**Current work**	**CTU-UHB**	**Balance** **(105/105)**	**RP+CNN**			**Acc: 98.69** **Se: 99.29** **Sp: 98.10** **AUC: 98.70**

*N, Normal; P, Pathological; QI, quality index; Fm, *F*-measure. FE, feature extraction; FS, feature selection; C, classifier. S1, morphological; S2, linear; S3, non-linear (the detailed features can be found in their publications). PRSA, phase rectified signal average; SVM, support vector machine; S-SVM, Sparse SVM; RF, Random forest. ST, statistical test (*p*-value); GE, grammatical evolution; LCA, latent class analysis. GM, generative model; NB, Naïve Bayes; DT, decision tree (C4.5). Other abbreviations can be found in the text. The best performance is indicated in bold.*

In this study, our proposed CAD system did not perform any feature transformation, which was embedded in the CNN model for signal classification simultaneously, representing the unique advantage of DL compared with conventional ML approaches. To the best of our knowledge, when tested on the same open-access CTG database, the proposed approach achieved better classification performance so far compared to all other related work in predicting fetal hypoxia: Acc = 98.69%, Se = 99.29%, Sp = 98.10%, especially considering similar studies using the 1-dimensional FHR signal and the CNN model (Comert and Kocamaz, 2018; Li et al., 2018; Petrozziello et al., 2018), as illustrated in Table 5. Obviously, the attractive result proves that the CNN algorithm moderately improves on the performance of published feature extraction based methods. The fundamental advantage of the proposed method relies on the convolutional layers in the deep architecture that provide distinctive local features to describe the input data. In this manner, the input data can be put in the proper class without needing any feature extraction and selection processes. The performances of the conventional shallow networks can be improved by feeding the networks with these local discriminative deep features. Therefore, this method can be adopted as an end-to-end learning method and we can conclude that CNN plays an important role in the field of automated FHR analysis, but requires further work.

In addition, EFM devices ensure continuous monitoring of the fetal hypoxia in antepartum as well as intrapartum periods. In clinical practice, the length of the FHR signals is frequently kept as between 10 and 30 min for providing a consistent interpretation. Nonetheless, there is not a gold standard for the length of the FHR time series in the computerized FHR analysis. The duration of the CTG test may vary according to the special conditions of the pregnant or fetus. In this work, we determined the ideal FHR length for fetal hypoxia detection as 13 min in accordance with the experimental result (Figure 10).

As for the CNN model, the effect of the convolutional network depth on its accuracy is another important issue. The number of convolutional layers and used filters affect the network success. The deeper networks need more hardware resources and more time for training. These types of networks have a great complex architecture. The FHR signals possess the high-level non-linear characteristic. For this reason, a deep model is required to catch hypoxia from FHR traces. After the extensive experimental setups (Table 4), we found the 8-layer deep CNN was quite efficient. Moreover, a CNN network can be trained from scratch if there is enough time, an available large-scale data source, and sufficient hardware, as in our previous experiment. However, in most of the “real world” problems, pretrained networks are configured for a new specific task using a transfer learning approach, since it is truly difficult or sometimes impossible to provide sufficient conditions to train a CNN network from scratch. Comert and Kocamaz (2018) used the transfer learning approach on a pretrained network called Alexnet, which was trained using 1.2 million images for 1000 classes. As a result, the authors reported promising results with Se of 56.15% and Sp of 96.51%.

In summary, the proposed CAD system has several attractive advantages:

(1) Feature extraction and selection techniques are not required;
(2) The 8-layer deep CNN is implemented and its parameters are analyzed to obtain optimal performance;
(3) Eight minutes is the minimum length of the FHR signal containing relevant information regarding the fetus, and 13 min achieves better performance using the CNN for fetal state classification.
(4) The system achieves better classification performance in predicting fetal hypoxia than other state-of-the-art methods.

## Conclusion

Fetal distress induced by hypoxia has become one of the main causes of neonatal death; therefore, its precise diagnosis can offer obstetricians an opportunity to intervene in a timely manner before damage occurs to the fetus. The FHR signal, part of the CTG, is routinely employed to monitor the fetal state during the antepartum and intrapartum stages. Unfortunately, visual interpretation of such signals is difficult for obstetricians since its special properties are irreproducible and subjective. Thus, CAD systems have been designed to analyze FHR signals automatically and assess the fetal state objectively in recent decades.

In this work, we proposed a novel CAD system to predict fetal hypoxia based on an RP and deep CNN. An open-access CTU-UHB database was used to test the performance and the umbilical artery pH was chosen as the gold standard to separate the fetal state into two classes. We randomly selected the same number of normal and pathological fetuses to avoid the influence of class imbalance. After signal preprocessing, the RP was adopted to reflect the non-linear characteristics of the FHR signal. The final image dataset consisted of 21,000 cases for each class by changing the values of three optional parameters of the RP. After comprehensive experiments on optimizing the CNN configuration, we obtained better performance: the Acc, Se, Sp, and AUC were 98.69, 99.29, 98.10, and 98.70%, respectively.

Unlike conventional ML approaches, our proposed system did not perform any complex feature engineering (i.e., feature extraction and selection). To the best of our knowledge, we achieved better classification performance in predicting fetal hypoxia using FHR signals compared with all other related work. In summary, the results proved the effectiveness of our proposed CAD system, which can assist obstetricians making objective medical decisions more accurately in clinical practice.

In the near future, we will test the performance of the proposed CAD system with more clinically collected data. Additionally, we plan to combine the FHR signal with other biomedical signals (e.g., UC) to improve accuracy when providing more reliable decision support tools.

## Data Availability

The database (CTU-UHB) for this study can be found in the PhysioNet (https://www.physionet.org/physiobank/database/ctu-uhb-ctgdb/).

## Author Contributions

ZZ and YZ contributed to the conception and design of the study. YZ performed the analysis and wrote the first draft of the manuscript. ZZ, ZC, and YD wrote sections of the manuscript. All authors contributed to manuscript revision, read and approved the submitted version.

## Conflict of Interest Statement

The authors declare that the research was conducted in the absence of any commercial or financial relationships that could be construed as a potential conflict of interest.
